# O_2_ Activation at an Enzymatic Diiron Site: Bridging Ligand Substitutions Alter Diferric‐(Hydro)peroxo States

**DOI:** 10.1002/anie.202519180

**Published:** 2025-12-26

**Authors:** Jae‐Hun Jeoung, Stefan Rünger, Kilian Weißer, Jakob Ruickoldt, Samriddhi Bhattacharya, Christian Limberg, Holger Dobbek

**Affiliations:** ^1^ Institute of Biology Humboldt‐Universität zu Berlin Philippstraße 13 10115 Berlin Germany; ^2^ Institute of Chemistry Humboldt‐Universität zu Berlin Brook‐Taylor‐Strasse 2 12489 Berlin Germany

**Keywords:** Bridging carboxylate ligands, Diiron, Dioxygen activation, Four‐helix bundle, Peroxo‐diferric intermediates

## Abstract

A variety of non‐heme diiron enzymes employ a conserved 2‐His‐4‐carboxylate motif to coordinate a dinuclear Fe site and activate dioxygen for diverse types of reactions. Two of the carboxylate residues act as bridging ligands between the Fe ions. As the type and coordination geometry of the bridging ligands in the diferrous state are thought to modulate reactivity, they were used to group diiron oxygenases into three structural subclasses. Here, we use the small diiron‐enzyme sulerythrin as a model to demonstrate that replacements of the bridging carboxylate amino acids allow us to decrease the distance between the two Fe ions, change the coordination of the bridging ligands from 1,3‐carboxylates to 1,1‐carboxylates and generate all three structural subclasses of diferrous active sites within the same protein scaffold. In addition to the known classes, we generated a coordination mode containing two 1,1‐carboxylate bridges. The resulting changes in the Fe coordination also alter the nature of the diferric (hydro)peroxo intermediates formed upon reaction with O_2_. Finally, we show that modulating the carboxylate bridges influences the reactivity of sulerythrin with O_2_. We establish sulerythrin as a versatile platform to engineer distinct diFe centers by a few exchanges, producing various stable (hydro)peroxo intermediates for further studies.

## Graphical abstract

Substituting the bridging carboxylates in diFe sulerythrin shifts the bridging ligand coordination from 1,3‐carboxylate to 1,1‐carboxylate, yielding all three diferrous bridging motifs in one protein. This shortens the Fe–Fe distance and increases reactivity with O_2_. O_2_ exposure triggers Fe rearrangements and traps two distinct diferric (hydro)peroxo intermediates.

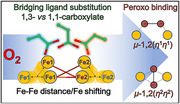

## Introduction

Oxygenases catalyze oxidative transformations in a wide variety of cellular processes by harnessing the reactivity of activated oxygen‐derived species to advance otherwise difficult to achieve reactions.^[^ ^1^, ^2^, ^3^, ^4^, ^5^ ^]^ Among them, non‐heme diiron (diFe)‐containing oxygenases use a dinuclear Fe site to mediate diverse processes, including iron storage,^[^ ^6^ ^]^ radical generation,^[^ ^7^ ^]^ and C─H bond hydroxylation.^[^ ^8^, ^9^ ^]^

A large group of these enzymes employs a conserved 2‐His‐4‐carboxylate ligand motif embedded in a four‐helix bundle (FHB) fold to coordinate the diFe site. Subtle structural variations in this coordination environment modulate reactivity and have been used to distinguish structural and functional subclasses.^[^ ^3^, ^4^, ^10^ ^]^ Starting from a diferrous state, these enzymes react with O_2_ proceeding through a series of well‐characterized intermediates. Early in this series, the peroxodiferric intermediate P is formed,^[^ ^3^, ^11^ ^]^ which, depending on its stability, may progress to intermediate Q, found in the soluble methane monooxygenase^[^ ^9^ ^]^ or to intermediate X, found in certain ribonucleotide reductases.^[^ ^3^, ^12^ ^]^ The nature, stability and protonation state of the (hydro)peroxo species^[^ ^11^ ^]^ determine if the peroxo‐diferric intermediate P functions as reactive oxidant or merely a precursor to the reactive high‐valent Fe species.

**Figure 1:**
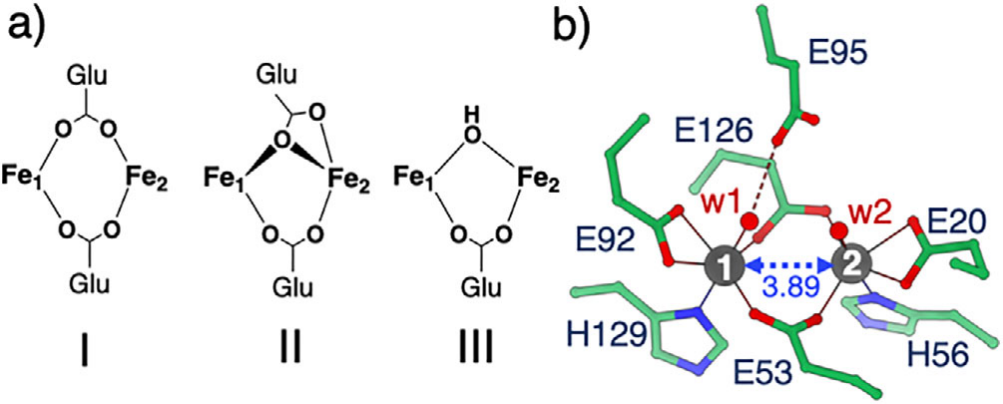
Three coordination geometries of the bridging ligands a) and the diferrous active site of wt‐SulE (PDB ID: 7O8A^[^ ^17^ ^]^) b). The three bridging coordination geometries shown in a) are: (I) Two 1,3‐carboxylate bridges, (II) one 1,1‐ and one 1,3‐carboxylate bridge and (III) one 1,3‐carboxylate bridge together with a (*μ*‐hydroxo)‐ligand. The Fe⋅⋅⋅Fe distance in b) is indicated by a blue‐dashed arrow and is given in Å. Water ligands are indicated by numeric w. Carbon (green), nitrogen (blue), and oxygen (red) are color‐coded.

Pivotal to the structure and reactivity of these intermediates are the bridging ligands between the Fe ions, whose nature and position modulate the Fe⋅⋅⋅Fe distance and metal accessibility, influencing substrate binding, and redox properties. Accordingly, diFe oxygenases have been classified into three subsets based on the coordination geometry of the bridging ligands: i) two 1,3‐carboxylate bridges, ii) one 1,1‐ and one 1,3‐carboxylate bridge, and iii) one 1,3‐carboxylate bridge together with a (*μ*‐hydroxo) ligand (Figure 1a).^[^ ^3^ ^]^ A dynamic rearrangement of the carboxylates between the different bridging modes, termed the carboxylate shift,^[^ ^13^ ^]^ can change the coordination environment, redox potentials, and access for O_2_ and substrates.^[^ ^2^, ^3^, ^13^, ^14^, ^15^, ^16^ ^]^

To investigate the structural and functional role of the carboxylate bridges for the reactivity of the diFe site, we used the small diFe enzyme sulerythrin (SulE) as a model system. SulE is structurally related to rubrerythrins,^[^ ^18^ ^]^ but lacks the auxiliary rubredoxin domain.^[^ ^19^ ^]^ SulE has been isolated from the aerobic, thermoacidophilic archaeon *Sulfololobus tokodaii*,^[^ ^19^, ^20^ ^]^ where it likely contributes to the detoxification of reactive oxygen species, by rapidly reducing hydrogen peroxide to water.^[^ ^17^ ^]^ SulE adopts a homodimeric architecture, in which each domain‐swapped monomer harbors a nearly symmetric diFe site coordinated by two EXXH sequence motifs with X referring to variable amino acid residues.^[^ ^17^ ^]^ In the diferrous SulE crystal structure (Figure 1b), the Fe ions have a slightly distorted trigonal bipyramidal coordination (*τ*_5_ = 0.93)^[^ ^21^, ^22^ ^]^ with two *syn*/*syn*‐bridging glutamates (Glu53 and Glu126),^[^ ^17^ ^]^ classifying SulE within the first diFe subset, comprising two 1,3‐carboxylate bridges (Figure 1a).

Here we show that replacing the two bridging glutamates residues in SulE, alters both the coordination environment and the Fe⋅⋅⋅Fe distance, enabling us to recreate all three classes of diferrous active sites as well as an additional coordination mode within the same protein fold. The exchanges open coordination sites and lead to the formation of diverse O_2_‐derived intermediates. Notably, we show that modulating the bridging residues can enhance the O_2_ reactivity of SulE, establishing it as a promising scaffold for engineering diFe based reactivity.

## Results and Discussion

### Protein Production and Metal Contents

SulE variants were heterologously expressed in their metal‐free (apo) form, exhibiting a minimal residual iron content (<0.2 mol Fe/mol protein), as previously reported.^[^ ^17^ ^]^ For all subsequent experiments, the apo‐SulE variants were reconstituted with iron under anoxic conditions using either the natural Fe isotope mixture or enriched ^57^Fe for Mössbauer spectroscopy. Iron reconstitution yielded an iron content exceeding 75% for all variants (Table S1).

### Mössbauer Spectroscopy of the Reduced Di^57^Fe‐Sites

**Figure 2:**
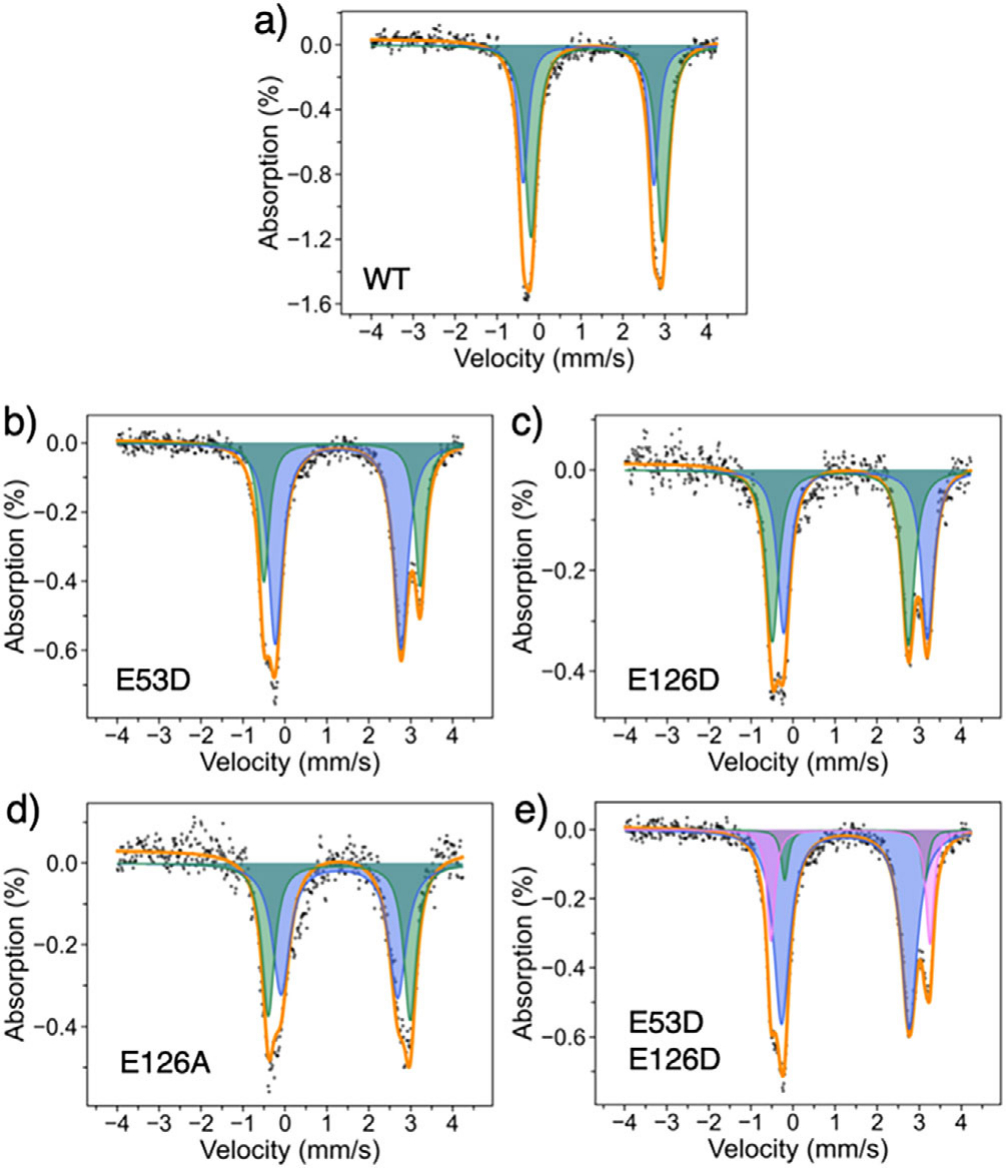
Zero‐field Mössbauer spectra at 14 K of ^57^Fe‐labelled SulE variants after reduction with sodium dithionite. a) wt‐SulE. b) E53D. c) E126D. d) E126A. e) E53D:E126D. While the empty circles represent the experimental spectral data, simulations of fitting are overlaid as solid orange lines. Fittings of sub‐spectra are shown with different colors: green, blue, and violet. For details see Table S2.

To probe the electronic and structural properties of the reduced diFe sites, we employed Mössbauer spectroscopy on the dithionite‐reduced diFe center of ^57^Fe‐SulE variants at 14 K in zero‐field (Figure 2 and Table 1). The data were analyzed using the fit that yielded the lowest *chi* ^2^ value. The Mössbauer spectra of all SulE samples showed as the major species high‐spin diFe(II) in their active site (Table 1). The isomer shifts and quadrupole splittings agree with a 5‐ or 6‐coordination by O/N ligands, as found for wild‐type SulE (wt‐SulE) (Figure 1).

**Table 1:** Mössbauer parameters for the SulE variants and the corresponding iron contents and energy levels.

Variant	Isomer shift *δ* (mm s^−1^)	Quadrupole splitting Δ*E*_Q_ (mm s^−1^)	Energy level	Oxidation state	Fe content (%)
Reduced state					
wt	1.18, 1.38	3.12, 3.14	High spin	Fe(II)	100 (38 + 62)
E126D	1.13, 1.49	3.24, 3.43	High spin	Fe(II)	100 (49 + 51)
E126A	1.30, 1.30	2.78, 3.38	High spin	Fe(II)	100 (54 + 46)
E53D	1.36, 1.27	3.72, 3.00	High spin	Fe(II)	100 (66 + 34)
E53D:E126D	1.12, 1.50, 1.36	3.20, 3.45, 3.08	High spin	Fe(II)	100 (46 + 31 + 23)
After reaction with O _2_					
Wt+O_2_	0.55	1.64	High spin	Fe(III)	100
E126D+O_2_	0.40, 0.59	0.90, 0.99	High spin	Fe(III)	100 (50 + 50)
E126A+O_2_	0.58, 0.38	0.86, 0.81	High spin	Fe(III)	100 (50 + 50)
E53D+O_2_	0.22, 0.59 1.62	1.13, 1.00 2.83	High spin High spin	Fe(III) Fe(II)	80 (40 + 40) 20
E53D:E126D+O_2_	0.52, 0.49	1.21, 0.73	High spin	Fe(III)	100 (50 + 50)

### Structures of the Reduced SulE Variants

**Figure 3:**
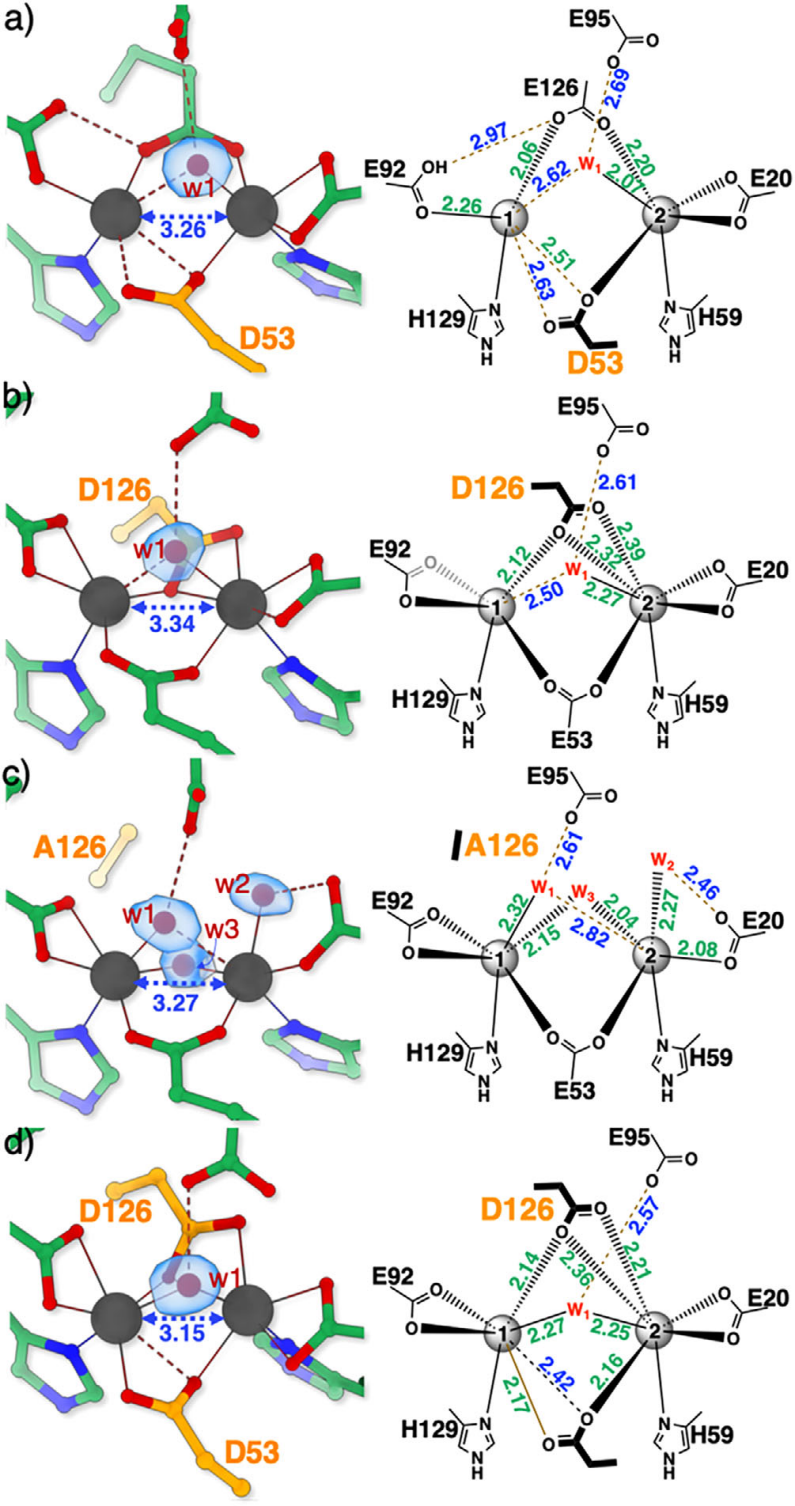
Crystal structures of reduced SulE variants: a) E53D, b) E126D, c) E126A, and d) E53D:E126D.  Substituted side chains are highlighted with orange carbon atoms (left panel). Right panels display selected bond lengths and interatomic distances. The deviations among crystallographically independent molecules are detailed in Figure S1. Iron atoms (dark‐gray spheres) are labeled in the right panel, and Fe⋅⋅⋅Fe distances are indicated by blue dashed arrows (in Å). Bond lengths and interatomic distances discussed in the main text are shown in Å. Further details are shown in Figure S1. Coordinated water molecules are labeled “w” followed by their identifier. *F*_obs_–*F*_calc_ ligand‐omit maps (contoured at 4 σ) are shown as blue surfaces for terminal or bridging ligands at the iron ions. SigmaA‐weighted 2*F*_obs_–*F*_calc_ maps are provided in Figure S2.

To investigate the effect of the bridging ligands on the diFe‐coordination environment, we replaced Glu53–one of the native *μ*‐1,3‐carboxylate ligands (Figure 1)–with the shorter aspartate side chain (E53D‐SulE). In contrast to Glu53, which coordinates both Fe ions as a symmetric *μ*‐1,3‐carboxylate, Asp53 adopts an asymmetric monodentate *μ*‐1,1‐carboxylate mode. In this arrangement, the carboxylate group interacts with Fe1 weakly in a bidentate manner (2.51 and 2.63 Å, Figures 3a and S1) and with Fe2 via a monodentate coordination. This altered geometry induces a small adjustment in the coordination of Glu126, resulting in a more planar bridging arrangement between the Fe ions compared to wt‐SulE (Figure 3a and Table S2). We modelled a mono‐atomic ligand near the Fe2 ion as a water molecule with an Fe─O bond length of about 2.07 Å, completing an octahedral coordination for Fe2. The water ligand is in hydrogen‐bonding distance to Glu95 (2.69 Å), resulting for Fe1 in a distorted trigonal pyramidal geometry with an open coordination site. Furthermore, the E53D exchange induces a repositioning of Glu92, which becomes a monodentate ligand for Fe1 and comes into hydrogen bonding distance to Glu126 (2.97 Å). Together, these rearrangements shorten the Fe⋅⋅⋅Fe distance by 0.65 Å compared to wt‐SulE, with a separation of 3.26 Å.

Glu126 is the second bridging residue, positioned *trans* to Glu53. When we exchanged it against aspartate (E126D‐SulE), we generated an analogous change to that observed for E53D‐SulE, but with inverted coordination around the two Fe ions. In E126D‐SulE, Asp126 bridges the diFe site in a *μ*‐1,1‐carboxylate mode, coordinating Fe1 monodentately and Fe2 in a bidentate mode (Figure 3b and Table S2). As in the E53D variant, a water molecule (w1) between the diFe center leaves Fe1 with an only weakly coordinated site, overall generating a distorted trigonal pyramidal coordination. Fe2 adopts a distorted trigonal bipyramidal geometry (*τ*_5_ = 0.90) with water serving as the fifth ligand. Throughout this work, carboxylates engaging in symmetric bidentate coordination are treated as single ligands for coordination geometry assignment.^[^ ^23^ ^]^ The E126D substitution results in a shortening of the Fe⋅⋅⋅Fe distance to 3.34 Å.

When we replaced Glu126 by the short hydrophobic amino acid alanine (E126A‐SulE), we observed further substantial changes to the active site (Figure 3c). The former position of the carboxylate of Glu126 is now occupied by a bridging water or *μ*‐hydroxo ligand (w3), with Fe─w3 bond lengths of 2.04 and 2.15 Å and an ∠Fe1–O^w3^–Fe2 of 101°, consistent with a *μ*‐hydroxo coordination.^[^ ^24^ ^]^ The Glu53 carboxylate and both Fe ions are nearly coplanar (Table S2). Two additional water molecules complete the coordination spheres of the two iron(II) centers. One water ligand (w1) binds Fe1 (2.32 Å) and is in H‐bond distance to Glu95 (2.61 Å), as also observed in wt‐SulE (Figures 1 and S3),^[^ ^17^ ^]^ supporting a terminal rather than a bridging role. A second water molecule (w2) coordinates Fe2 and is further stabilized by a short hydrogen bond (2.46 Å) from Glu20. Notably, Glu20 switches from bidentate (in wt‐SulE) to monodentate coordination of Fe2. The overall changes preserve a trigonal bipyramidal coordination for Fe1 (*τ*_5_ = 1.07), while Fe2 adopts a square pyramidal geometry (*τ*_5_ = 0.05) with one open coordination site. The Fe⋅⋅⋅Fe distance in the E126A variant is 3.27 Å, slightly shorter than in E126D‐SulE.

When we exchanged both bridging glutamate residues against aspartate (E53D:E126D), we induced two significant structural changes at the diFe site (Figure 3d). The coordination modes of Asp53 and Asp126 closely resemble those in the respective single variants: Asp53 acts as a bidentate ligand to Fe1, and Asp126 coordinates Fe2 bidentately. Both residues bridge Fe1 and Fe2 via monodentate *μ*‐1,1‐carboxylate interactions. A single water molecule (w1) bridges the Fe ions symmetrically (2.27 Å to Fe1 and 2.25 Å to Fe2). These modifications yield the shortest observed Fe⋅⋅⋅Fe distance among the variants, 3.15 Å, approximately 0.8 Å shorter than in wt‐SulE. Notably, the double exchange creates an additional coordination mode not covered by the known three subclasses (Figure 1a).^[^ ^3^ ^]^

### Oxygen Reaction Kinetics of the SulE Variants

**Figure 4:**
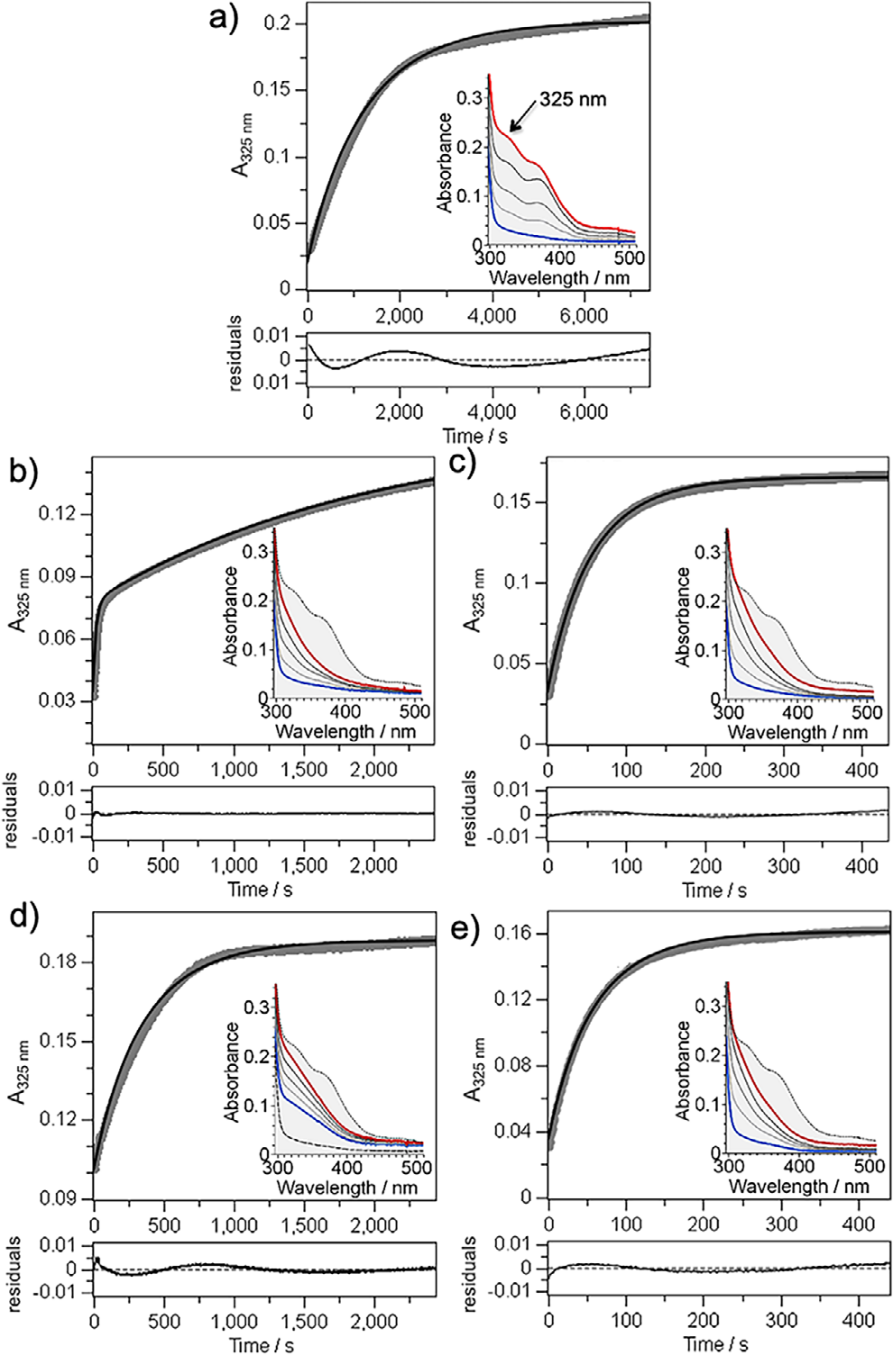
Kinetic traces at 325 nm and UV–vis spectra of reduced diFe‐SulE variants after reaction with O_2_. a) wild type. b) E53D. c) E126D. d) E126A. e) E53D:E126D. Absorbance changes at 325 nm are shown as thick gray lines; global fits obtained with DynaFit 4^[^ ^27^ ^]^ are overlaid as thin black lines. Residuals of the fit are shown in the corresponding lower panels. Unless stated otherwise, model 1 was used for analysis (see Materials and Methods for details). All data were fitted by monophasic kinetics, except for E53D‐SulE. Insets display UV–vis spectral changes in the 300–550 nm range in the course of reacting with O_2_; the 551–750 nm region is omitted due to the absence of relevant features. Spectral transitions are indicated by solid lines, progressing from the initial reduced state (blue) via intermediate states (gray), to the oxidized state (red). For comparison, the spectrum of oxidized wt‐SulE is shown as a gray‐filled area under a dotted‐line. In panel d, the reduced spectrum of wt‐SulE is shown as a dashed‐line.

We monitored the reactivity of the diFe(II)‐SulE variants with O_2_ by UV–vis spectroscopy (Figure 4). To generate the reduced state for the subsequent reaction with O_2_, we used ascorbic acid instead of sodium dithionite to avoid direct reactions between the reductant and O_2_. In the reduced state, wt‐SulE has a nearly featureless spectrum between 300 and 400 nm. With the exception of the E126A variant, all SulE variants with substituted bridging glutamate(s) showed in the reduced state the same featureless spectrum (insets, blue lines in Figure 4b,e). Upon exposure with O_2_, wt‐SulE developed three distinct absorption shoulders at 325, 375, and 470 nm (inset, Figure 4a), closely resembling the spectrum observed previously for wt‐SulE after reacting with an excess of H_2_O_2_.^[^ ^17^ ^]^ Notably, we did not detect intense ligand‐to‐metal charge transfer (LMCT) bands at longer wavelengths (500–700 nm), such as those typically associated with the peroxo‐to‐Fe(III) transitions in other non‐heme diiron enzymes, including MMOH, RNR, hemerythrin, and desaturases.^[^ ^3^, ^25^, ^26^ ^]^ In contrast, O_2_ exposure of the SulE variants resulted in an increase in absorbance, but without the distinct shoulders observed for wt‐SulE, indicating formation of a different type of initial product or its decay (insets, Figure 4b–e). To evaluate these differences, we analyzed the kinetic traces at 325 nm (Figure 4) using two kinetic models (Scheme S1). Model 1 describes a single second‐order reaction between the protein and O_2_. Model 2 includes a sequential mechanism, in which an O_2_‐reacted intermediate is converted to a final species. These models might reflect, for example, the reduction of the bound O_2_ or a structural rearrangement at the metal site. Fits were performed using Dynafit 4,^[^ ^27^ ^]^ and models selected based on global fitting statistics (see Supporting Information for details). For all SulE variants except E53D‐SulE, the simpler second‐order reaction best described the data.

Wt‐SulE exhibited a very slow reaction with O_2_ (*k*_1 _= 3.1 M^−1^s^−1^). In contrast, all variants with bridging glutamate substitutions reacted significantly faster (Table S3 and Figure S3a). The E53D variant showed the highest initial rate constant (*k*_1_ = 188.8 M^−1^s^−1^), but the kinetic trace required the inclusion of a second, slower phase (*k*_2_ = 1.26 × 10^−3^ s^−1^), indicating the accumulation of an intermediate that slowly converted to the final state. In contrast, the E126A variant exhibited the lowest reactivity toward O_2_ among all variants (*k*_1 _= 11.4 M^−1^s^−1^) (Table S3). Surprisingly, the E53D:E126D variant displayed a similar reactivity (*k*_1_ = 67.5 M^−1^s^−1^) as E126D (*k*_1_ = 70.8 M^−1^s^−1^), suggesting no additive effect from the second substitution. Although we only modelled a second, slower phase for the E53D variant, we cannot exclude very slow rearrangement processes (e.g., *k* of 10^−9^ s^−1^ and slower) occurring in other variants.

In all variants, the rate constant *k*_1_ increased compared to wt‐SulE (Figure S3a), reflecting enhanced reactivity with O_2_. Absorbance changes at 325 nm were of similar magnitude across all variants (Figure S3b), except for E126A, where the amplitude of change was approximately 50% compared to wt‐SulE. For E53D‐SulE, the dominant absorption change (Δ*ε*_2_) was associated with formation of the final state, whereas the contribution of the intermediate state (Δ*ε*_1_) was smaller.

### Mössbauer Spectra of the O_2_‐Oxidized DiFe Sites

**Figure 5:**
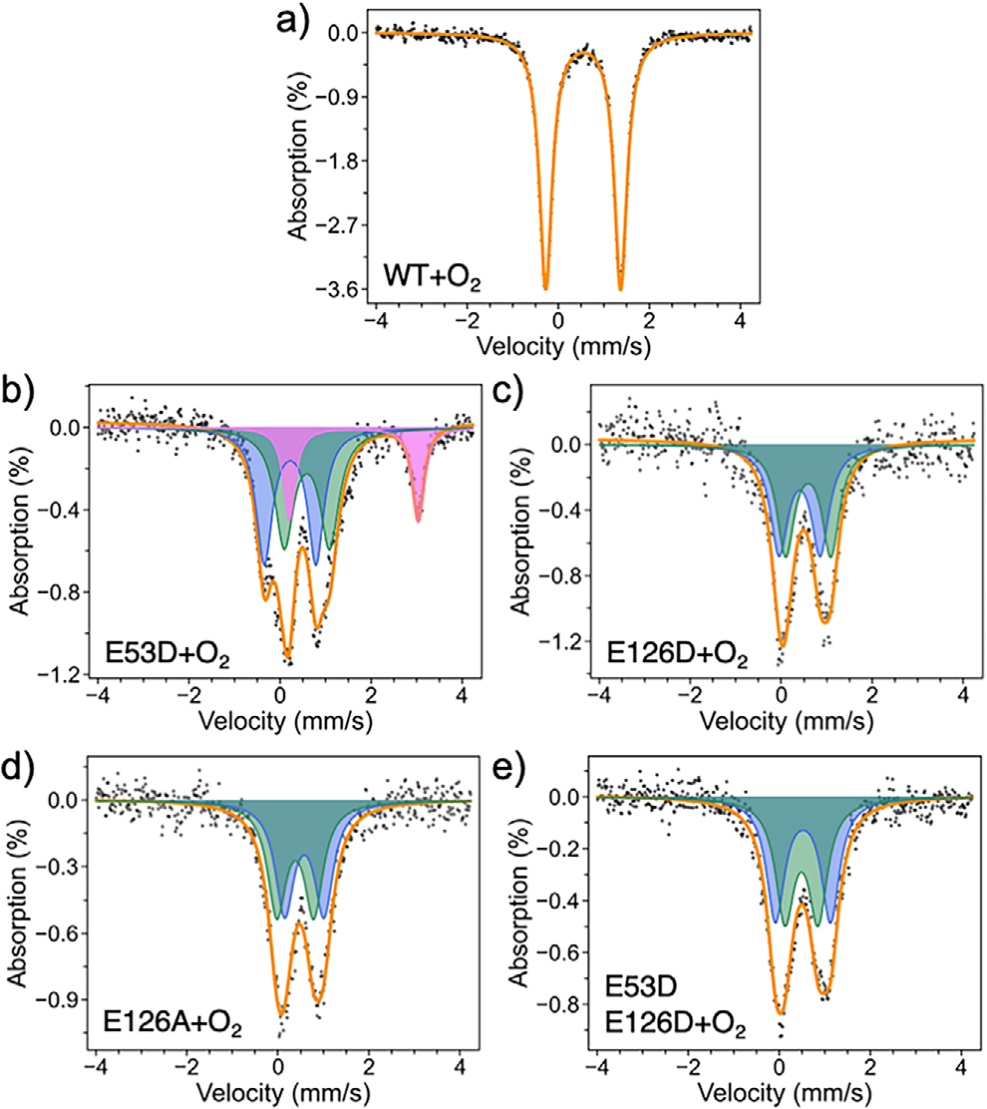
Zero‐field Mössbauer spectra at 14 K of ^57^Fe‐labelled SulE variants reacted with dioxygen: a) wt‐SulE+O_2_, b) E53D+O_2_, c) E126D+O_2_, d) E126A+O_2_, e) E53D:E126D+O_2_. While the empty circles represent the experimental spectral data, simulations of fitting are overlaid as solid orange lines. Fitting of sub‐spectra components are shown with different colors: green, blue, and violet.

As UV/Vis spectroscopy gives only indirect evidence on the outcome of reactions between the SulE variants and O_2_, we also used Mössbauer spectroscopy to analyze the products of the reaction between O_2_ and the ^57^Fe‐labelled SulE variants (Figure 5 and Table 1).

The diFe site of the ^57^Fe‐SulE wild type was completely converted into high‐spin Fe(III) after exposure to molecular oxygen, as indicated by a single doublet with Mössbauer parameters *δ* = 0.55 mm s^−1^ and Δ*E*_Q_ = 1.64 mm s^−1^ (Figure 5a), similar to the oxo‐bridged resting centers of the stearoyl‐acyl carrier protein Δ9‐desaturase.^[^ ^28^, ^29^ ^]^ The Mössbauer spectra of ^57^Fe‐labelled SulE variants (E126D, E126A, and E53D:E126D) after O_2_ exposure exhibited isomer shifts ranging from 0.38 to 0.59 mm s^−1^ and quadrupole splittings between 0.73 and 1.21 mm s^−1^. These parameters indicate that the diiron ions have been oxidized to high‐spin diFe(III) moieties, characterized by two overlapping doublets with a 1:1 ratio (Figure 5c–e). The Mössbauer spectrum of the E53D variant exhibited three quadrupole doublets (Figure 5b). The two major species, accounting for 80% of the total Fe, had isomer shifts of 0.22 and 0.59 mm s^−1^ with similar quadrupole splittings (Δ*E*_Q_ = 1.13 and 1.00 mm s^−1^), characteristic of high‐spin diFe(III) moieties (Table 1). The third doublet, representing 20% of the total Fe, displayed an isomer shift of 1.62 mm s^−1^ and a quadrupole splitting of 2.83 mm s^−1^, indicating the presence of a high‐spin Fe(II) species.

### Crystal Structures of the O_2_‐Incubated DiFe Sites of the SulE Variants

**Figure 6:**
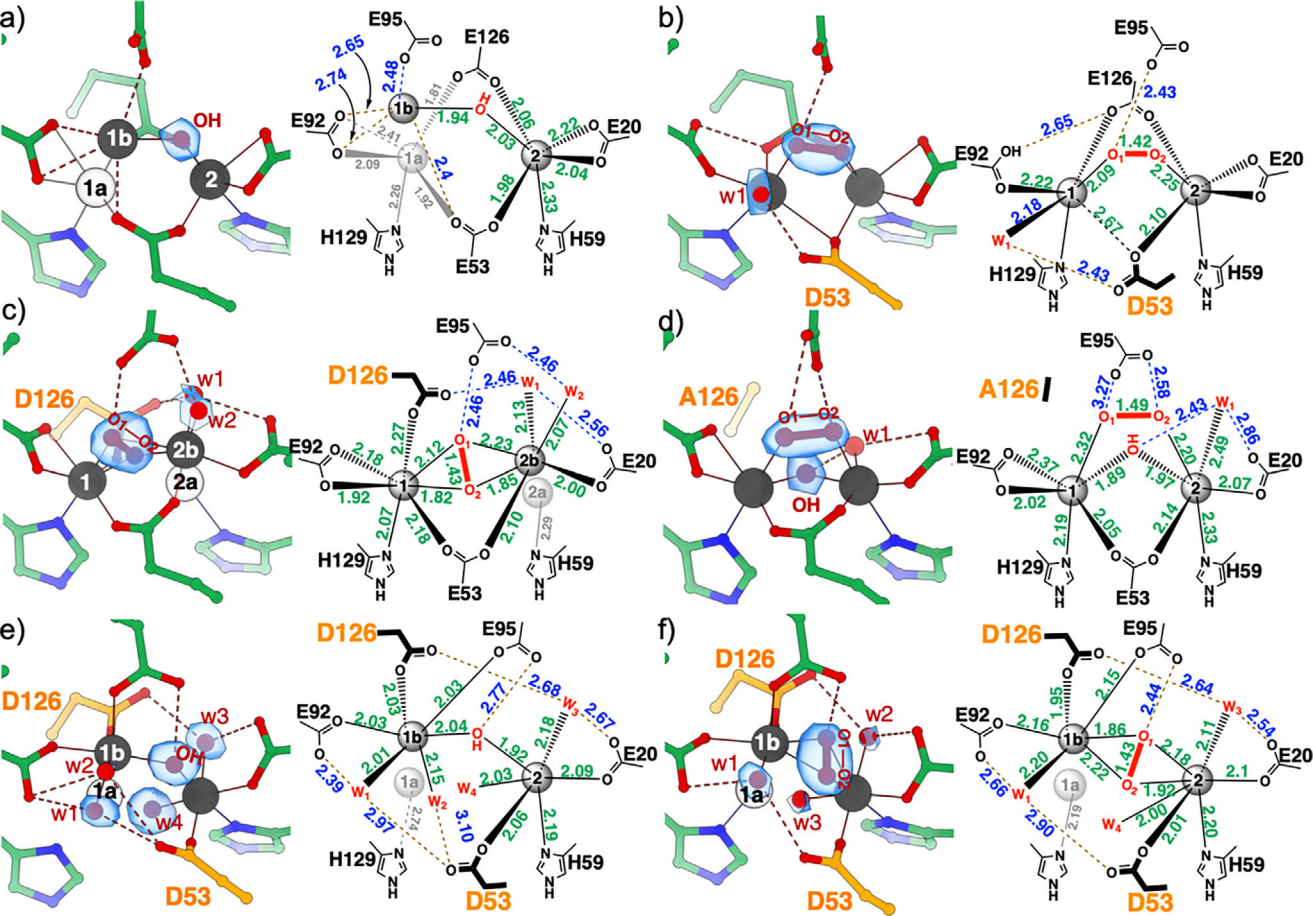
Crystal structures of O_2_‐reacted SulE variants. a) wt‐SulE+O_2_. b) E53D+O_2_. c) E126D+O_2_. d) E126A+O_2_. e) (hydro)oxo‐bridged and f) peroxo‐bridged forms of the E53D:E126D+O_2_ variant. Substituted side chains are highlighted with orange carbon atoms. Right panels display selected bond lengths and interatomic distances, with deviations from crystallographically independent molecules provided in Figure S1. Iron atoms (dark‐gray spheres) are numbered in panel a) and b) with alternative positions a) and b). Peroxide ligands are depicted as ball‐and‐stick models; O─O bond lengths were refined without restraints and given in Å. Bond lengths and distances discussed in the text are shown in Å. For further details see Figure S1. Coordinated water molecules are labelled with numbered “w” identifiers. *F*_obs_–*F*_calc_ ligand‐omit difference maps are contoured at 4 sigma, except 3 sigma for water (w) in b), c), d), and e), and shown as transparent blue surfaces. Anomalous Fourier difference and a sigma‐weighted 2*F*_obs_–*F*_calc_ maps are provided in Figure S2.

To gain structural insight into the product of the reaction of the SulE variants with O_2_, we determined the crystal structures of the diFe‐SulE variants after exposing the crystals to O_2_ (Figure 6).

In wt‐SulE, O_2_ exposure induces a larger rearrangement of the diFe site. While Fe2 (69% occupancy) remains in its original position, Fe1 partially shifts toward Glu95, forming a weak interaction in the newly occupied position (Fe1b, 58%; Fe1a, 40% occupancy). This shift is clearly supported by anomalous difference maps that reveal both iron positions (Figures 6a and S2; Table S4). Such iron movement upon oxidation has been observed previously for SulE^[^ ^30^ ^]^ and other FHB proteins including rubrerythrin and symerythrin.^[^ ^31^, ^32^, ^33^ ^]^ We observed electron density consistent with a *μ*‐hydroxide between Fe1b and Fe2, with Fe─O distances of 1.94 and 2.03 Å, and an occupancy matching that of Fe1b (58%). Glu92 coordinates Fe1a monodentately and remains in a short distance to Fe1b (2.65 and 2.74 Å) (Figures 6a and S1). The Fe⋅⋅⋅Fe distances contract upon oxidation to 3.49 Å (Fe1a⋅⋅⋅Fe2) and 3.33 Å (Fe1b⋅⋅⋅Fe2), both significantly shorter than in the reduced state (Figure 1).

In contrast to wt‐SulE, the E53D‐SulE variant retains its Fe positions after O_2_ exposure. We observed a strong elliptical omit density at a bridging position between the diFe sites (Figure 6b), which we modeled as a diatomic peroxide species and refined without bond restraints to an O─O bond length of 1.43 Å – consistent with a *cis μ*‐1,2(*η* ^1^ *η* ^1^)‐(hydro)peroxo ligand. Fe─O bond lengths are slightly longer than in synthetic analogues of unprotonated peroxo ligands,^[^ ^3^ ^]^ consistent with a protonation. A short hydrogen bond between the peroxide and Glu95 (2.43 Å), indicates proton sharing. The ligand geometry (114° for ∠Fe1–O1–O2; 107° for ∠Fe2–O2–O1 and 39° for the dihedral angle ∠Fe1–O1–O2–Fe2) is comparable to characterized non‐heme Fe(III)‐(hydro)peroxo complexes.^[^ ^34^, ^35^ ^]^ The coordination mode of the protein‐derived ligands remains largely unchanged compared to the reduced state, except for Asp53, whose non‐bridging carboxylate oxygen is now in short hydrogen‐bonding distance (2.43 Å) to a new water ligand on Fe1 (Figures 6b and S1). The Fe⋅⋅⋅Fe distance hardly changed upon oxidation (3.29 Å).

The E126D variant undergoes a substantial rearrangement at the diiron site upon O_2_ incubation (Figures 6c and S2). Fe1 retains its position (79% occupancy), while Fe2 splits between two conformers (Fe2a:36% occupancy; Fe2b:64% occupancy) (Table S4). A strong diatomic electron density between the two Fe sites was refined without bond restraints as a unique side‐on *μ*‐1,2(*η* ^2^ *η* ^2^)‐(hydro)peroxo ligand with an O─O bond length of 1.43 Å. The (hydro)peroxo ligand is asymmetrically bound with one oxygen closer to Fe1 and Fe2b (1.82 and 1.85 Å), while the other is positioned further away (2.12 and 2.23 Å). The ligand has bond angles of 58° (∠Fe1–O1–O2) and 87° (∠Fe2–O2–O1) comparable to other Fe(III)‐peroxo species,^[^ ^10^ ^]^ with a unique dihedral angle (∠Fe1–O1–O2–Fe2) of 130°. Fe1 adopts a trigonal bipyramidal geometry (*τ*_5_ = 1.05) with O1_peroxo_ and Nδ^His129^ as axial ligands. Asp126 switches from bridging to monodentate coordination on Fe1, while Glu20, formerly bidentate, becomes monodentate on Fe2b. Both carboxylates form short hydrogen bonds (2.46–2.56 Å) with newly introduced water ligands (w1, w2), which also interact with Glu95. These changes correlate with a contracted Fe1–Fe2b distance of 3.20 Å. The Fe2a site and corresponding 3.39 Å Fe–Fe separation match the reduced structure, likely representing an unreacted enzyme fraction.

O_2_ exposure of E126A‐SulE alters the coordination of the diiron site, without shifting the Fe positions (Figure 6d and S2). A spherical electron density between the metals was modeled as a bridging *μ*‐OH ligand (Fe─O = 1.89 Å/1.97 Å; ∠Fe1–*μ*‐OH–Fe2 = 112°). An elliptical omit density refined to a *cis μ*‐1,2(*η* ^1^ *η* ^1^)‐peroxo ligand (O─O = 1.49 Å; 69 ± 6% occupancy) (Figure 6d), consistent with the Fe2 occupancy at 56 ± 3%, while Fe1 is at 81% (Table S4). The slightly longer Fe─O_peroxo_ bonds (>2.2 Å) suggest protonation,^[^ ^3^, ^36^ ^]^ consistent with a hydroperoxide assignment.^[^ ^24^, ^37^, ^38^ ^]^ Both Fe ions are hexacoordinate.

Crystals of E53D:E126D‐SulE revealed two distinct O_2_‐derived states within the asymmetric unit (Figure 6e,f). Fe1 predominantly occupies the Fe1b position (78%), while Fe2 remains unchanged (90%). In two copies, a round‐shaped electron density was modeled as a bridging *μ*‐OH ligand (Figure 6e). In the third copy, we found an elliptical electron density that we refined without bond restraints as a side‐on *μ*‐1,2(*η* ^2^ *η* ^2^)‐(hydro)peroxo‐ligand (O─O = 1.43 Å, Fe─O = 1.86–2.22 Å) (Figure 6f), with angles closely resembling that of the O_2_‐reacted E126D variant. In the *μ*‐OH bound state, Fe1b is ligated by Glu92, Glu95, Asp126, two water molecules (w1, w2), and the *μ*‐OH bridge; Fe2 is coordinated by His56, Glu20, two water molecules (w3, w4), and the *μ*‐OH. In the peroxo‐bound state, coordination is preserved, with the hydroxo bridge replaced by the (hydro)peroxo ligand. Glu95 supports both *μ*‐OH and (hydro)peroxo species via H‐bonds (2.77 and 2.44 Å, respectively). The Fe⋅⋅⋅Fe distance in oxidized E53D:E126D‐SulE are 3.47 Å (*μ*‐OH) and 3.31 Å (peroxo), both slightly longer than in the reduced state, consistent with the observed ligand rearrangement and the incorporation of additional solvent molecules.

### Comparing Wild‐Type SulE with Variants Containing Exchanged Bridging Ligands

Our starting point was the structure of reduced wt‐SulE, which features a *syn*‐*syn μ*‐1,3‐carboxylate bridging ligand structure formed by Glu53 and Glu126, keeping the two Fe(II) ions at a distance of 3.89 Å (Figure 1).^[^ ^17^ ^]^ The Mössbauer parameters confirm two high‐spin Fe(II) ions, in agreement with the observed O/N coordination environment and previous XAS data, which indicated 70% ferrous and only 30% ferric iron for as‐reconstituted wt‐SulE.^[^ ^17^ ^]^ In this study, the presence of the reducing agents ascorbate and sodium dithionite ensured complete conversion to the ferrous state.

While diferrous wt‐SulE reacts rapidly with hydrogen peroxide,^[^ ^17^ ^]^ its reaction with O_2_ is very slow, consistent with a physiological role in hydrogen peroxide reduction rather than O_2_ activation. Upon O_2_ exposure, the two high‐spin Fe(II) ions are slowly oxidized to high‐spin Fe(III). Interestingly, despite the homogeneous appearance in Mössbauer spectra, the crystal structure of oxidized wt‐SulE reveals two conformations for Fe1. The reasons for this structural heterogeneity are not fully understood. Although partial photoreduction cannot be entirely excluded, it is unlikely under the low X‐ray dose conditions used (0.57 MGy), which was sufficient to preserve the oxidation state of the mixed valence (Fe(II)/Fe(III)) species.^[^ ^30^ ^]^ Notably, the Fe1a–Fe2 distance is 0.4 Å shorter than in the reduced state, primarily due to a movement of Fe1a, indicating that Fe1a is not a photoreduced species, but rather represents a structurally distinct conformation of the oxidized state.

Replacing either of the bridging glutamate ligands (Glu53 or Glu126) with aspartate preserved the high‐spin diferrous state, with slight variations in the isomer shifts and quadrupole splittings, indicating altered diFe coordination. Both E53D‐ and E126D‐SulE variants induced a switch from the  two 1,3‐carboxylate to the 1,1‐ and 1,3‐carboxylate bridge type in the reduced state,^[^ ^3^ ^]^ wherein the remaining glutamate retained its original coordination, while the aspartate formed an asymmetric *μ*‐1,1‐carboxylate bridge. This conversion in coordination mode upon removal of a methylene unit from the side chain was reproducible and independent of which glutamate was substituted.

Despite their structural similarity in the reduced state, the two variants exhibit markedly different O_2_ reactivities. While both reacted faster than wt‐SulE, E53D‐SulE showed a substantially higher rate enhancement.

Mössbauer spectroscopy revealed further distinctions: E126D‐SulE behaved comparably to other oxidized variants with high‐spin Fe(III) ions, whereas E53D‐SulE was more heterogeneous, including a ferrous component. This suggests incomplete oxidation, possibly due to accumulation of a mixed‐valent Fe(II, III)‐O_2_* intermediate (S‐state) that converts slowly to a (hydro)peroxo‐bound diFe(III) form.

The two variants also stabilized distinct oxidized species: E126D‐SulE formed a side‐on *μ*‐1, 2(*η* ^2^ *η* ^2^)‐bound (hydro)peroxo complex accompanied by Fe2 movement, while E53D‐SulE retains its Fe positions and stabilized a *cis μ*‐1,2(*η* ^1^ *η* ^1^)‐(hydro)peroxo species.

The structure of the double exchange E53D:E126D‐SulE in the reduced state combines the effects of the two individual exchanges. Upon O_2_ exposure, however, its reactivity more closely resembled the slower‐reacting E126D‐SulE, both in kinetic behavior and Mössbauer parameters. The two different species found in oxidized crystals (Figures 6 and S1) may represent sequential intermediates along a reaction pathway, with the side‐on *μ*‐1,2(η^2^:η^2^)‐(hydro)peroxo species preceding the *μ*‐hydroxo species. However, such a transformation would require protonation and two additional electrons. While photoreduction is a possible electron source, it seems unlikely given the absence of an analogous progression in other variants. Alternatively, protonation of the hydroperoxo ligand may have generated H_2_O_2_, which subsequently dissociated and created space for water binding and hydroxo formation. Notably, crystallographic trapping of multiple intermediate states within a single asymmetric unit has been reported for other oxygenases,^[^ ^39^, ^40^, ^41^ ^]^ possibly due to crystal packing effects stabilizing distinct conformations along a reaction pathway.

In E126A‐SulE, replacement of Glu126 against alanine eliminates one bridging carboxylate and opens coordination sites on both Fe ions. This geometry favors a bridging (*μ*‐hydroxo)‐ligand together with a 1,3‐carboxylate in the reduced state, characteristic of the third diFe oxygenase subclass.^[^ ^3^ ^]^ Upon reacting with O_2_, E126A‐SulE stabilizes a *cis μ*‐1,2(η^1^η^1^)‐(hydro)peroxo‐ligand. Despite the short Fe⋅⋅⋅Fe distance and the fact, that the Fe⋅⋅⋅Fe distance is almost retained upon reacting with O_2_ under peroxide formation, E126A‐SulE reacts only marginally faster with O_2_ than wt‐SulE. Thus, Fe⋅⋅⋅Fe proximity alone does not dictate reactivity.

Once reacting with O_2_, the E126A‐SulE forms a *cis μ*‐1,2(η^1^η^1^)‐(hydro)peroxo complex similar to that in E53D‐SulE, despite differing first coordination spheres. Therefore, different changes of Glu126 produce distinct diferric‐(hydro)peroxide species, highlighting the absence of a simple correlation between substitution type and intermediate geometry.

**Figure 7:**
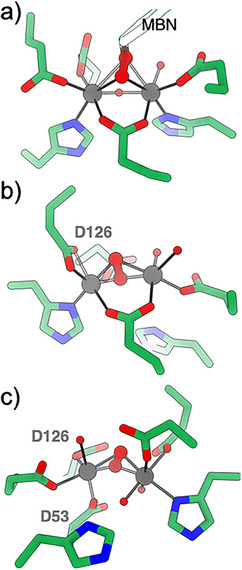
Comparison of structures with side‐on bound peroxide ligand. a) The oxygenated toluene bound T4moHD (PDB ID: 5TDT^[^ ^10^ ^]^). The toluene moiety (MBN) is shown as a transparent model. The SulE variants of b) E126D and c) E53D‐E126D. For clarity, Fe2a in b) and Fe1a in c) are not shown. Three structures are similarly oriented.

In our study, the nature of the (hydro)peroxo ligand correlates with the mobility of the Fe centers. In E53D‐ and E126A‐SulE, where both Fe ions remain coordinated to their respective histidines (in *trans* position to the peroxo ligand), the oxidized species adopt a *cis μ*‐1,2(η^1^η^1^)‐(hydro)peroxo geometry. In contrast, *μ*‐1,2(η^2^η^2^)‐peroxo binding is observed when one Fe ion detaches from its His ligand, as in E126D and the (hydro)peroxo state of the double variant. Both Fe ions appear capable of undergoing this rearrangement (Figures 6 and S1). The presence of multiple (hydro)peroxo species is noteworthy, as they likely differ in reactivity. In the toluene 4‐monooxygenase complex (T4moHD), three distinct peroxo‐diferric intermediates were observed in crystals: *μ*‐1,2(η^1^η^1^)‐peroxo, *μ*‐1,1‐(hydro)peroxo, and *μ*‐1,2(η^2^η^2^)‐peroxo species.^[^ ^10^, ^36^ ^]^ Among these, only the *μ*‐1,2(η^2^η^2^)‐peroxo diferric complex is catalytically competent.^[^ ^10^ ^]^ The *μ*‐1,2(η^2^η^2^)‐peroxo diferric complex of T4moHD is very similar to the *μ*‐1,2(η^2^η^2^)‐peroxo diferric complexes of SulE, including the position of water molecules (Figure 7).

## Conclusions

Our study demonstrates that subtle modification of the bridging carboxylates can systematically alter the coordination geometry and O_2_ reactivity of a diFe site. However, no simple correlation was found between Fe⋅⋅⋅Fe distance, extent of rearrangement, and the type of (hydro)peroxo intermediate formed or the rate of reaction with O_2_. Moreover, despite some enhancement of O_2_ reactivity, all engineered SulE variants remain slow compared to native diferrous oxygenases, which exhibit first‐order rate constants in the range of 10–1000 s^−1^,^[^ ^3^ ^]^ whereas the approximate first order rate constants of the fastest SulE variants is below 0.1 s^−1^. This suggests that second coordination sphere interactions play a decisive role in reactivity tuning. For wt‐SulE and its variants it is likely that Glu95 (Figure 1) plays an important role, which consistently forms (very) short hydrogen‐bonds to the (hydro)peroxo ligands. Such interactions may stabilize these intermediates and prevent progression to higher‐valent iron species. Further studies are required to determine whether the stabilized (hydro)peroxo species can be harnessed for substrate oxidation, and which additional modifications could transform a peroxide converting enzyme to an efficient artificial oxygenase.

## Supporting Information

The authors have cited additional references within the Supporting Information. (References [42, 43, 44, 45, 46, 47, 48, 49, 50, 51, 52])

## Conflict of Interests

The authors declare no conflict of interest.

## Supporting information



Supporting Information

## Data Availability

All coordinates and structure factor amplitudes are available in the Protein Data Bank (PDB) under the PDB‐IDs: 9S4P, 9S3W, 9S4C, 9S5V, 9S59, 9S4R, 9S66, 9S5D, 9S4O.
