# Supplementary material for: O2 Activation at an Enzymatic Diiron Site: Bridging Ligand Substitutions Alter Diferric‐(Hydro)peroxo States
Source: Angew Chem Int Ed Engl. 2025 Dec 26;65(5):e19180. doi: 10.1002/anie.202519180 (PMC12851003; doi:10.1002/anie.202519180)
Supplement: Supplementary file 1 — Supporting Information [file ANIE-65-e19180-s001.docx]

Supplementary Information

O_2_ Activation at an Enzymatic Diiron Site: Bridging Ligand Substitutions Alter Diferric~~-~~(Hydro)peroxo States

Jae-Hun Jeoung^+[a]^, Stefan Rünger^+[a]^, Kilian Weißer^[b]^, Jakob Ruickoldt^[a]^, Samriddhi Bhattacharya^[a]^ Christian Limberg^[b]^, Holger Dobbek*^[a]^

[a] Dr. Jae-Hun Jeoung^+^, Dr. Stefan Rünger^+^, Dr. Jakob Ruickoldt, Samriddhi Bhattacharya, Prof. Dr. Holger Dobbek*
Institute of Biology
Humboldt-Universität zu Berlin
Philippstraße 13, 10115, Berlin, Germany

E-Mail: [holger.dobbek@hu-berlin.de](mailto:holger.dobbek@hu-berlin.de)

[b] Kilian Weißer, Prof. Dr. Christian Limberg
Institute of Chemistry
Humboldt-Universität zu Berlin
Brook-Taylor-Strasse 2, 12489, Berlin, Germany

[+] These authors contributed equally to this work.

**Experimental Details**

**Materials**.

Preparations of anoxic and metal-free buffers were as previously published.^[17]^ Anoxic works of Fe-reconstitution and crystallization were conducted under an atmosphere of 95% N2 /5% H2 inside a glovebox (model B; COY Laboratory Products, Inc.).

**Protein production.**

The quick-change site-directed method was applied to exchange single amino acids of SulE. DpnI-digested PCR-amplified DNA product was transformed into *Escherichia coli* DH5α. After plasmid preparation, positive variants were confirmed by DNA sequencing (Eurofins Genomics Germany GmbH). Apo-SulE variants were expressed and purified as described previously.^[17]^ The protein concentration was determined by the Bradford method using bovine serum albumin as a standard.

**Metal contents analysis.**

We used a colorimetric assay with 1,10-phenantroline^[42]^ to determine the metal content of SulE samples as reported previously.^[17]^

**Preparation of ^57^Fe^2+^**

The ^57^Fe solution was prepared as follows. 33.5 mg ^57^Fe powder was weighed into 10 ml clean glass vials (Hungate tube) and incubated overnight with 1 ml 8 M HCl at room temperature. The vial was covered with parafilm during this process to allow H_2_ to escape. After overnight incubation, 4 ml of H_2_O was added, followed by 200 mg of tri-sodium citrate dihydrate (C_6_H_5_Na_3_O_7_ x 2 H_2_O). The solution was transferred to a 15 ml tube and carefully adjusted to pH 5-6 with 5% ammonia solution.

**Fe reconstitution.**

All metal reconstitutions were conducted under anoxic conditions in the glovebox. Apo-SulE (500 μM) in buffer A (50 mM Tris-HCl pH 7.5 and 150 mM NaCl) was mixed with a metal solution of 1.3 mM FeCl_2_ (or the prepared ^57^Fe solution for reconstituting with ^57^Fe) with 2 mM ascorbic acid to produce mildly reducing conditions. The metal-protein mixture was incubated for 18 h at 18°C. Excess free metals were removed using a spin concentrator by several cycles of dilution/concentration (>144-fold dilution with buffer A).

**^57^Fe-enriched sample preparation for Mössbauer spectroscopy.**

The content of ^57^Fe was also determined by the phenanthroline colorimetric assay.^[42]^ Protein samples for Mössbauer spectroscopy were adjusted to a concentration of approximately 1 mM in the anoxic glove box in buffer A, resulting in a ^57^Fe concentration of 1.6-1-9 mM (ranging from 80-95% depending on the SulE variants). The ^57^Fe-SulE samples were reduced using 5 mM Na-dithionite for 5 min, then transferred to the sample holder for Mössbauer spectroscopy, directly frozen and stored in liquid N_2_ until measurement. To generate the oxygen-reacted state the ^57^Fe-SulE samples were thawed under atmospheric conditions, pressurized at 1.25 bar of pure oxygen (99.9%) for 2 hours at 22°C in an in-house gas chamber, transferred to the sample holder and frozen in liquid N_2_.

**Mössbauer spectroscopy.**

Mössbauer spectra were recorded at 14 K with a *SeeCo* MS6 spectrometer equipped with a *Janis* CCS-850 cryostat and a *CTI-Cryogenics* 8200 helium compressor. Temperature control was achieved with a *LakeShore* 335 thermocontrol. The samples were loaded under inert conditions at 77 K using a helium counterflow setup.

**Oxidation kinetics of Fe-SulE variants.**

The diFe-SulE variants were reduced under anoxic conditions in the glove box. For this purpose, samples were incubated with ascorbic acid for 24 h at a molar ratio of 1:2 of protein:ascorbic acid. Excess ascorbic acid was removed using a PD10 column (Sephadex G-25, Sigma-Aldrich). The reduced diFe-SulE (50 µM) was pipetted into air-saturated buffer A under thorough stirring for complete mixing. The buffer was previously gassed with compressed air for 5 min so that the oxygen concentration was approximately 271 µM, calculated assuming the percentage of O_2_ in the atmosphere to be 20.9% and using a Henry constant of 1.3 mM/atm.^[43]^ The change in absorption spectra, especially at 325 nm, was monitored using an Agilent 8453 spectrophotometer. Experiments were performed in a quartz cuvette with a stirrer, at 25°C.

The extent of the absorption changes was determined by fitting the absorbance at 325 nm to exponential functions. For most samples, a monoexponential function was used: y = A × (1 - e^-^*^k^*^t^) + d. For the E53D variant, the data were better described by a biexponential function: y = A_1_ × (1 - e^-^*^k^*^1t^) + A_2_ × (1 - e^-^*^k^*^2t^) + d, where *y* represents the absorbance, *t* is time, *k* is the rate constant, *A* is the amplitude, and *d* is the offset.


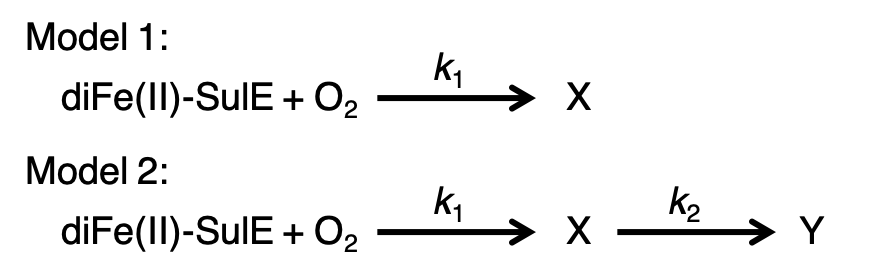


**Scheme S1**. Two alternative models applied to the reaction of diFe(II)-SulE variants with O_2_. Model 1 describes the reaction as an irreversible second-order process generating state X. Model 2 extends model 1 by including an irreversible conversion of X to Y. Kinetic data were analyzed with Dynafit 4^[27]^ using both models, which were expressed as a set of ordinary differential equations (ODE) and numerically solved to fit the reaction traces. Fits were performed with the “trust-region” algorithm of DynaFit 4. We determined confidence intervals (95% probability) for the best-fit parameters using the profile-t method. All reaction traces were evaluated with both models, and the built-in model discrimination routine of DynaFit was applied to assess the quality of fit. Although model 2 consistently resulted in lower residuals, it was rejected for all variants except E53D, as parameter variations, mostly for *k*_2_, were unacceptably large.

ODEs for model 1:

*d*[E]/*d*t = -*k*_1_[E][O_2_]

*d*[O_2_]/*d*t = -*k*_1_[E][O_2_]

*d*[X]/*d*t = *k*_1_[E][O_2_]

ODEs for model 2:

*d*[E]/*d*t = -*k*_1_[E][O_2_]

*d*[O_2_]/*d*t = -*k*_1_[E][O_2_]

*d*[X]/*d*t = *k*_1_[E][O_2_]-*k*_2_[X]

*d*[Y]/*d*t = *k*_2_[X]

**Crystallization and post-crystallization treatments.**

The crystals of diFe-SulE variants were obtained by sitting-drop vapor diffusion under anoxic conditions in the glovebox, as described previously, in a condition containing 0.1 M Bis-Tris pH 5.5 and 20-25% (w/v) polyethylene glycol 3350 by mixing with 13 mg/mL protein with 2 mM ascorbic acid in 1:1 ratio.^[17]^ Crystals were flash cooled in liquid N_2_ with 15% (v/v) 2*R*,3*R*-butanediol as cryoprotectant to obtain the reduced state structures. For O_2_ incubation, the crystals were transferred to an in-house build gas chamber and pressurized at 1.25 bar of pure oxygen (99.9%) for 2 hours at 22°C. The oxygen treated crystals were flash cooled as described for the reduced state crystals.

**Data collection, structure determination, and refinement.**

Diffraction data were collected at beamline BL 14.1 (BESSY-II, Helmholtz-Zentrum Berlin Adlershof) at 100 K using an energy of 13.5 keV.^[44]^ Anomalous scattering of iron was recorded at 7140 eV to calculate anomalous Fourier maps. Data were collected with different transmission at 13.5 keV: reduced crystals were measured at 100% transmission (4.25 · 10^10^ photons/s), whereas oxygen-incubated crystals were measured with 10% beam flux (2.73 · 10^9^ photons/s) using a 50 µm aperture. Diffraction data were indexed, integrated, and scaled with XDSAPP^[45,46]^, which uses XDS.^[47]^ Initial phases were obtained from a structure located using Patterson search techniques with AutoMR from Phenix^[48]^ using PDB ID: 7O8A^[17]^ as a search model. After iterative model building using Coot,^[49]^ further refinements were performed using phenix.refine (Phenix) and refmac5.^[48,50]^ No restraints were applied to the metal-metal distances of the diiron sites or the O-O distances of the peroxo species in any of the variants during refinement. Initial attempts to model the peroxo species using a single water molecule or an oxygen atom resulted substantial residual electron density, suggesting the presence of a diatomic species. This assignment was further supported by the elliptical shape of the observed electron density. Modeling with two water molecules was also unsatisfactory due to too short interatomic distances. Protonation states of oxygen-containing bridging ligands were inferred from Fe-O bond length, with 1.73 Å – 1.82 Å assigned to *μ*-oxo and 1.96 – 2.06 Å to *µ*-hydroxo species.^[24]^ To more precisely determine ligand–metal distances, ligand-omitted *F*_o_–*F*_c_ electron-density maps were calculated (Fig. 1 and Fig. 6). The resulting clear positive density allowed unambiguous placement of bridging ligands and refinement without geometric restraints. The terminal or bridiging water molecules were modeled when the distance lies between 2.00 and 2.27 Å. Bond distances for all ligands are shown in Fig. S1, with deviations calculated based on the number of independent molecules in the crystallographic asymmetric unit.

Occupancies of Fe atoms in all reported structures exceeded 80 – 100% for diferrous sites and 53 – 100% for diferric sites (Table S4). Data collection and refinement statistics are given in Table S5. Coordinates and structure factor amplitudes have been deposited in the Protein Data Bank under the PDB accession codes listed in Table S5.

**Other software.**

All structural figures in this report were generated using UCSF ChimeraX version 1.8.^[51]^ Spectra and kinetic traces were plotted using Matplotlib with Python 3.11.8.^[52]^

**Supplementary Tables**

**Table S1.** Fe-content determination.

| Sample | wt | E126D | E126A | E53D | E53D  :E126D | E95D | E95A |
| --- | --- | --- | --- | --- | --- | --- | --- |
| ^56^Fe/Protein (mol/mol) | 2.0 ± 0.2 | 1.8 ± 0.1 | 1.6 ± 0.1 | 1.6 ± 0.1 | 1.7 ± 0.2 | 2.0 ± 0.2 | 2.0 ± 0.1 |
| ^57^Fe/Protein (mol/mol) | 1.9 ± 0.1 | 1.7 ± 0.1 | 1.8 ± 0.1 | 1.5 ± 0.2 | 1.7 ± 0.2 | 1.8 ± 0.1 | 1.7 ± 0.1 |

**Table S2.** Dihedral angles of selected atoms. Angle deviations are calculated from the number of copies in the asymmetric unit (n). Angles are in degree (°).

|  | Fe1-O1^126^-O2^126^-Fe2 | Fe1-O1^53^-O2^53^-Fe2 | Fe1-O1^Peroxo^-O2^Peroxo^-Fe2 |
| --- | --- | --- | --- |
| Reduced | | | |
| WT (n = 3) | 42 ± 1 | 43 ±3 |  |
| E53D (n = 6) | -6 ± 3 | 70 ± 2 |  |
| E126D (n = 2) | 71 ± 3 | 36 ± 2 |  |
| E126A (n = 2) |  | -5 ± 1 |  |
| E53D-E126D (n = 2) | 63 ± 1 | 61 ± 1 |  |
| O2-reacted | | | |
| WT (n = 6) | 40 ± 7 | -11 ± 7 |  |
| E53D (n = 2) | -13 ± 1 | 70 ± 6 | 39 ± 9 |
| E126D (n = 3) | 30 ± 3 | -8 ± 6 | 130 ± 17 |
| E126A (n = 6) |  | -32 ± 7 | -11 ± 4 |
| E53D-E126D  -OH ligand (n = 2) | 69 ± 6 | 26 ± 1 |  |
| E53D-E126D  -OO ligand  (n = 1) | 70 | 20 | 120 |

**Table S3.** Rate constants for SulE variants at wavelength of 325 nm, determined by DynaFit using the model in Scheme S1.

| SulE variants | rate constant *k*_1_ (M^-1^ x s^-1^) | rate constant *k*_2_  (s^-1^) |
| --- | --- | --- |
| wt | 3.2 |  |
| E53D | 188.8 | 0.5 10^-3^ |
| E126D | 70.8 |  |
| E126A | 11.4 |  |
| E53D:E126D | 67.5 |  |

**Table S4.** Iron and ligand occupancies determined by crystallography. Values are shown as a percentage. SD: standard deviation. Average occupancies of iron and ligand are indicated by a green highlighting for the major species and a grey highlighting for the minor species.

|  |  | **Iron** | **contents** |  | **Bridging** | **ligand** |
| --- | --- | --- | --- | --- | --- | --- |
|  | ***1a*** | ***1b*** | ***2a*** | ***2b*** | ***Peroxide*** | ***Oxo*** |
| **WT+O_2_** | 38 | 62 | 61 |  |  | 58 |
|  | 42 | 58 | 69 |  |  | 58 |
|  | 47 | 53 | 71 |  |  | 58 |
|  | 48 | 52 | 63 |  |  | 58 |
|  | 55 | 45 | 68 |  |  | 58 |
|  | 42 | 58 | 68 |  |  | 58 |
| average | 45 | 55 | 67 |  |  | 58 |
| SD | 6 | 6 | 4 |  |  | 0 |
| **E53D** | 60 |  | 100 |  |  |  |
|  | 70 |  | 100 |  |  |  |
|  | 57 |  | 100 |  |  |  |
|  | 65 |  | 100 |  |  |  |
|  | 64 |  | 100 |  |  |  |
|  | 73 |  | 100 |  |  |  |
| average | 65 |  | 100 |  |  |  |
| SD | 6 |  | 0 |  |  |  |
| **E53D+O_2_** | 55 |  | 100 |  | 74 |  |
|  | 58 |  | 100 |  | 68 |  |
| average | 57 |  | 100 |  | 71 |  |
| SD | 2 |  | 0 |  | 4 |  |
| **E126A** | 82 |  | 100 |  |  |  |
|  | 91 |  | 85 |  |  |  |
| average | 87 |  | 93 |  |  |  |
| SD | 6 |  | 11 |  |  |  |
| **E126A+O_2_** | 78 |  | 56 |  | 68 |  |
|  | 81 |  | 60 |  | 75 |  |
|  | 100 |  | 58 |  | 60 |  |
|  | 75 |  | 53 |  | 65 |  |
|  | 77 |  | 55 |  | 68 |  |
|  | 76 |  | 54 |  | 75 |  |
| average | 81 |  | 56 |  | 69 |  |
| SD | 9 |  | 3 |  | 6 |  |
| **E126D** | 96 | 100 | 93 | 100 |  |  |
|  | 83 | 100 | 87 | 100 |  |  |
| average | 90 | 100 | 90 | 100 |  |  |
| SD | 9 | 0 | 4 | 0 |  |  |

*Table S4 continued*.

|  |  | **Iron** | **contents** |  | **Bridging** | **ligand** |
| --- | --- | --- | --- | --- | --- | --- |
|  | ***1a*** | ***1b*** | ***2a*** | ***2b*** | ***Peroxide*** | ***Oxo*** |
| E126D+O_2_ | 77 |  | 38 | 60 | 85 |  |
|  | 77 |  | 34 | 66 | 80 |  |
|  | 84 |  | 35 | 67 | 75 |  |
| average | 79 |  | 36 | 64 | 80 |  |
| SD | 4 |  | 2 | 4 | 5 |  |
| E53DE126D | 100 |  | 100 |  |  |  |
|  | 100 |  | 100 |  |  |  |
| average | 100 |  | 100 |  |  |  |
| SD | 0 |  | 0 |  |  |  |
| E53DE126D+O_2_ | 15 | 85 | 90 |  |  | 90 |
|  | 30 | 70 | 80 |  | 80 |  |
|  | 15 | 80 | 100 |  |  | 80 |
| average | 20 | 78 | 90 |  |  | 85 |
| SD | 9 | 8 | 10 |  |  | 7 |

**Table S5.** Crystallographic statistics on data collection and refinement.

| Crystal name | E53D_red | E126D_red | E126A_red | E53D:E126D_red |
| --- | --- | --- | --- | --- |
| PDB-ID | 9S4P | 9S3W | 9S4C | 9S5V |
| **Data collection** | | | | |
| Wavelength (Å) | 0.91841 | 0.91841 | 0.91841 | 0.91841 |
| Space group | *C*2 | *P*6_3_ | *P*6_3_ | *P*6_3_ |
| Cell dimensions |  |  |  |  |
| a b c (Å) | 117.56 88.68 100.99 | 74.20 74.20 100.60 | 72.72 72.72 99.29 | 72.56 72.56 98.89 |
| α β γ  (°) | 90.0 114.86 90.0 | 90.0 90.0 120.0 | 90.0 90.0 120.0 | 90.0 90.0 120.0 |
| Resolution (Å) | 45.81-1.45 (1.54-1.45) | 39.61-1.44 (1.52-1.44) | 38.99-1.46 (1.55-1.46) | 36.28-1.18 (1.25-1.18) |
| No. reflections total  /unique | 1121071  /164726 | 378430  /56840 | 262778  /51701 | 479063  /96602 |
| Redundancy | 6.81 | 6.66 | 5.08 | 4.96 |
| *R*_meas_ | 6.5 (105.1) | 7.8 (156.9) | 8.0 (138.9) | 5.6 (212.4) |
| *I* / s*I* | 16.35 (1.82) | 11.22 (0.83) | 11.94 (1.05) | 14.54 (0.71) |
| *CC_1/2_* | 99.9 (71.5) | 99.9 (56.3) | 99.8 (99.3) | 100.0 (30.6) |
| ISa | 27.87 | 15.36 | 23.19 | 33.14 |
| Completeness (%) | 98.8 (97.6) | 99.7 (98.6) | 99.9 (44.6) | 99.8 (99.1) |
| **Refinement** | | | | |
| Reflections used | 164723 | 56840 | 51701 | 96601 |
| *R*_work_ / *R*_free_ | 16.5/20.2 | 15.1/19.5 | 14.9/19.5 | 13.4/16.4 |
| Ramachandran statistics |  |  |  |  |
| favored/allowed/outlier | 97.37/2.46/0.00 | 98.20/1.80/0.00 | 97.87/2.13/0.00 | 98.23/2.92/0.00 |
| R.m.s. deviations |  |  |  |  |
| Bond lengths (Å) | 0.012 | 0.007 | 0.005 | 0.013 |
| Bond angles (°) | 1.861 | 0.748 | 0.725 | 1.829 |

Values in parentheses are for the highest-resolution shell. red, reduced.

*Table S5 continued*.

| Crystal name | E53D+O_2_ | E126D+O_2_ | E126A+O_2_ | E53D:E126D+O_2_ |
| --- | --- | --- | --- | --- |
| PDB-ID | 9S59 | 9S4R | 9S66 | 9S5D |
| **Data collection** | | | | |
| Wavelength (Å) | 0.91841 | 0.91841 | 0.91841 | 0.91841 |
| Space group | *P*6_3_ | *C*2 | *P*2_1_ | *C*2 |
| Cell dimensions |  |  |  |  |
| a b c (Å) | 72.15 72.15 98.26 | 100.48 87.87 59.73 | 59.66 87.58 93.12 | 100.51    87.93    60.15 |
| α β γ  (°) | 90.0 90.0  120.0 | 90.0 115.85  90.0 | 90.0 101.01 90.0 | 90.0 115.11  90.0 |
| Resolution (Å) | 45.0-1.76  (1.81-1.76) | 48.88-1.75 (1.80-1.75) | 48.69-1.83  (1.88-1.83) | 45.76-1.7  (1.74-1.70) |
| No. reflections total  /unique | 595037  /28731 | 319893  /46697 | 568725  /81103 | 169324  /50392 |
| Redundancy | 20.71 | 6.85 | 7.01 | 3.36 |
| *R*_meas_ | 18.0 (336.0) | 5.7 (160.2) | 10.6 (181.9) | 4.7 (69.3) |
| *I* / s*I* | 13.97 (0.90) | 17.47 (1.27) | 11.21 (1.00) | 14.45 (1.81) |
| *CC_1/2_* | 99.9 (48.4) | 100.0 (66.6) | 99.9 (56.0) | 99.9 (84.0) |
| ISa | 24.63 | 30.14 | 24.56 | 24.15 |
| Completeness (%) | 100.0 (100.0) | 99.0 (99.5) | 97.8 (96.9) | 96.6 (97.9) |
| **Refinement** | | | | |
| Reflections used | 28731 | 46675 | 81103 | 50392 |
| *R*_work_ / *R*_free_ | 16.75/20.83 | 18.53/22.84 | 16.61/21.31 | 17.64/22.32 |
| Ramachandran statistics |  |  |  |  |
| favored/allowed/outlier | 97.16/2.84/0.00 | 91.05/8.96/0.00 | 97.60/2.40/0.00 | 97.61/2.39/0.00 |
| R.m.s. deviations |  |  |  |  |
| Bond lengths (Å) | 0.010 | 0.011 | 0.010 | 0.009 |
| Bond angles (°) | 0.970 | 1.046 | 0.952 | 0.968 |

Values in parentheses are for the highest-resolution shell.

*Table S5 continued*.

| Crystal name | wild type+O_2_ | E53D+O_2_  Fe-anomalous | E126D+O_2_  Fe-anomalous | E53D:E126D+O_2_ Fe-anomalous |
| --- | --- | --- | --- | --- |
| PDB-ID | 9S4O |  |  |  |
| **Data collection** | | | | |
| Wavelength (Å) | 0.91841 | 1.736 | 1.736 | 1.736 |
| Space group | *C*2 | *P*6_3_ | *C*2 | *C*2 |
| Cell dimensions |  |  |  |  |
| a b c (Å) | 117.90    88.23   100.24 | 72.15 72.15 98.26 | 100.48 87.87 59.73 | 100.51    87.93    60.15 |
| α β γ  (°) | 90.0 114.56  90.0 | 90.0 90.0 120.0 | 90.0 115.85 90.0 | 90.0 115.11  90.0 |
| Resolution (Å) | 48.7-1.55  (1.59-1.55) | 49.17-1.93 (2.04-1.93) | 48.69 - 2.00 (2.07-2.00) | 45.76 - 1.80  (1.86-1.80) |
| No. reflections total  /unique | 909550  /132125 | 421550  /43203* | 434335  /62305* | 139345  /42416* |
| Redundancy | 6.88 | 9.76 | 7.0 | 3.3 |
| *R*_meas_ | 9.3 (105.1) | 15.8 (293.0) | 8.9 (74.6) | 4.8 (51.3) |
| *I* / s*I* | 11.9 (2.1) | 10.6 (0.6) | 13.4 (2.4) | 14.3 (2.2) |
| *CC_1/2_* | 99.8 (76.8) | 99.9 (29.6) | 99.9 (87.7) | 99.9 (88.8) |
| ISa | 12.46 | 29.97 | 27.87 | 23.13 |
| Completeness (%) | 97.7 (97.1) | 99.8 (98.9) | 98.0 (98.0) | 96.7 (96.8) |
| **Refinement** | | | | |
| Reflections used | 132055 |  |  |  |
| *R*_work_ / *R*_free_ | 17.57/20.25 |  |  |  |
| Ramachandran statistics |  |  |  |  |
| favored/allowed/outlier | 98.21/1.79/0.00 |  |  |  |
| R.m.s. deviations |  |  |  |  |
| Bond lengths (Å) | 0.009 |  |  |  |
| Bond angles (°) | 0.970 |  |  |  |

* Friedel pairs are treated as different reflections. Values in parentheses are for the highest-resolution shell.

*Table S5 continued*.

|  | wild type+O_2_  Fe-anomalous |
| --- | --- |
|  |  |
| **Data collection** | |
| Wavelength (Å) | 1.736 |
| Space group | *C*2 |
| Cell dimensions |  |
| a b c (Å) | 117.90    88.23   100.24 |
| α β γ  (°) | 90.0 114.56  90.0 |
| Resolution (Å) | 48.83-1.92 (2.04-1.92) |
| No. reflections total/unique | 221103/65037* |
| Redundancy | 3.4 |
| *R*_meas_ | 10.4 (204.5) |
| *I* / s*I* | 8.09 (0.54) |
| *CC_1/2_* | 99.7 (28.7) |
| ISa | 20.84 |
| Completeness (%) | 91.9 (80.4) |
|  |  |

* Friedel pairs are treated as different reflections. Values in parentheses are for the highest-resolution shell.

**Supplementary Figures**


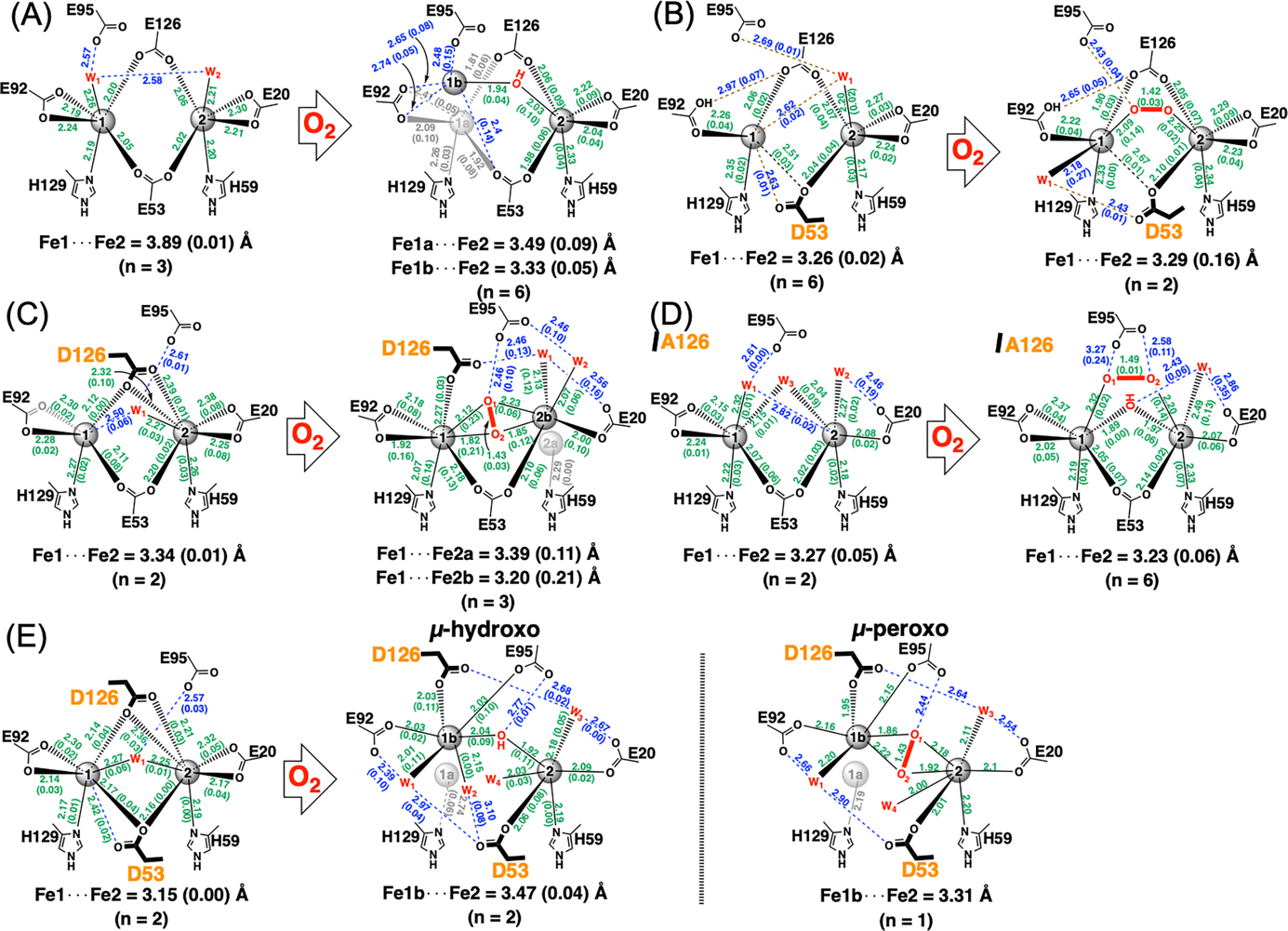


**Figure S1.** Schematic representation of bond lengths in reduced and O_2_-reacted SulE structures. (A) Wild type, (B)E53D, (C)E126D, (D)E126A, (E) hydroxo-bridged and peroxo-bridged states of the E53D:E126D variant. Hydrogen-bonding interactions are shown as blue dashed lines. Bond distances (in Å) represent average values derived from crystallographically independent diiron sites with identical coordination geometries within the asymmetric unit. Standard deviations (in parentheses) were calculated from the respective number of independent sites (n). The reduced wild-type structural data is taken from PDB ID: 7O8A.^[17]^


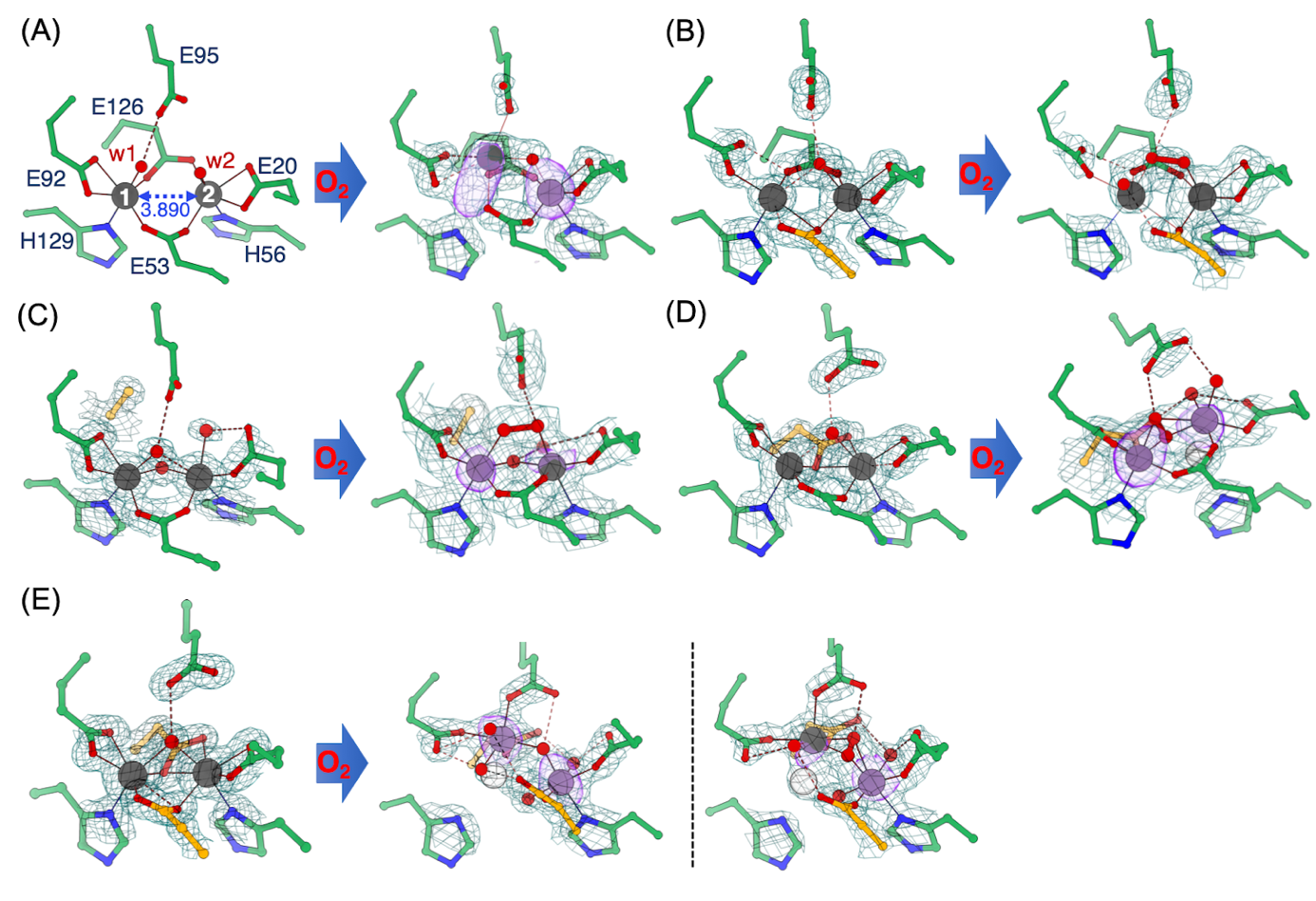


**Figure S2.** Crystal structures of the diiron sites in SulE variants with electron density maps for the reduced and the oxygen-reacted structures. (A) Wild type, (B)E53D, (C)E126D, (D)E126A, (E) hydroxo-bridged and peroxo-bridged states of the E53D:E126D variant. Mutated residues are shown with orange carbon atoms. Minor occupancy iron positions are depicted as transparent spheres. SigmaA-weighted 2*F*_obs_ – *F*_calc_ electron density maps are contoured at 2.0 σ (light blue mesh) and iron anomalous difference maps collected at 7140 eV are contoured at 5 σ (violet surface). The reduced wild-type structure is taken from PDB ID: 7O8A.^[17]^


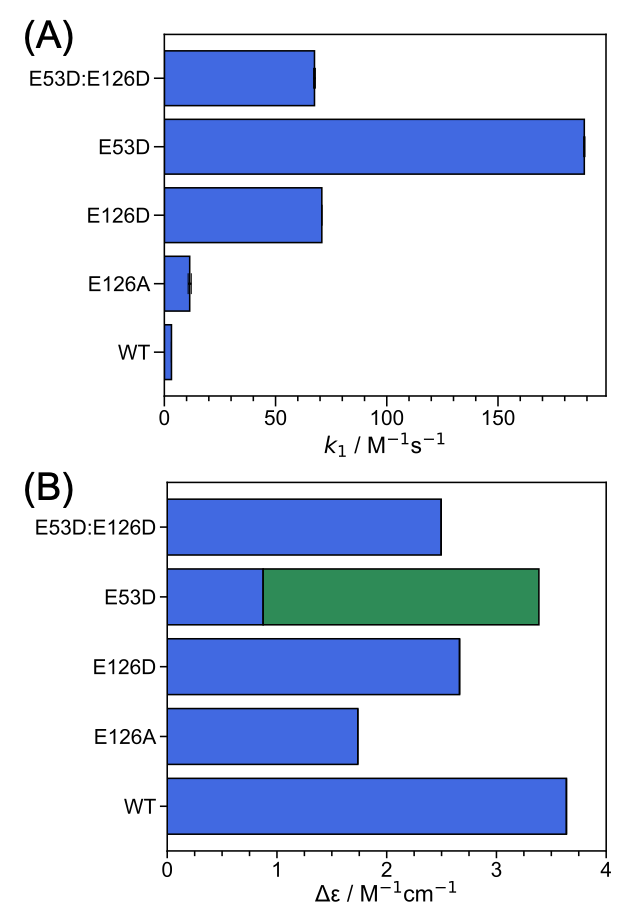


**Figure S3.** Kinetic parameters of the reaction of the diiron-SulE variants with O_2_. (A) Second order rate constant (*k*_1_) for the reaction with O_2_. Error bars represent 95% confidence intervals. (B) Difference molar absorption coefficients (Δε) of O_2_-derived species of the different diiron-SulE variants at 325 nm. For the E53D variant, Δε values of both the intermediate state (blue; Δε_1_) and the final state (green; Δε_2_) are shown. Confidence intervals fall within the line width of the column outlines and are therefore not displayed. The reaction of E53D-SulE does not follow simple second-order kinetics but requires a model incorporating an intermediate state (see Model 2 in Scheme S1).

**References**

[17] J.-H. Jeoung, S. Rünger, M. Haumann, B. Neumann, F. Klemke, V. Davis, A. Fischer, H. Dau, U. Wollenberger, H. Dobbek, “Bimetallic Mn, Fe, Co, and Ni Sites in a Four-Helix Bundle Protein: Metal Binding, Structure, and Peroxide Activation” *Inorg. Chem.* **2021**, *60*, 17498.

[27] P. Kuzmič, “Program DYNAFIT for the Analysis of Enzyme Kinetic Data: Application to HIV Proteinase” *Anal. Biochem.* **1996**, *237*, 260.

[42] B. B. Buchanan, W. Lovenberg, J. C. Rabinowitz, “A Comparison of Clostridal Ferredoxins” *Proc. Natl. Acad. Sci.* **1963**, *49*, 345.

[43] R. Sander, “Compilation of Henry’s Law Constants (version 4.0) for Water as Solvent” *Atmospheric Chem. Phys.* **2015**, *15*, 4399.

[44] U. Mueller, R. Förster, M. Hellmig, F. U. Huschmann, A. Kastner, P. Malecki, S. Pühringer, M. Röwer, K. Sparta, M. Steffien, M. Ühlein, P. Wilk, M. S. Weiss, “The Macromolecular Crystallography Beamlines at BESSY II of the Helmholtz-Zentrum Berlin: Current Status and Perspectives” *Eur. Phys. J. Plus* **2015**, *130*, 141.

[45] M. Krug, M. S. Weiss, U. Heinemann, U. Mueller, “XDSAPP: a Graphical User Interface for the Convenient Processing of Diffraction Data Using XDS” *J. Appl. Crystallogr.* **2012**, *45*, 568.

[46] K. M. Sparta, M. Krug, U. Heinemann, U. Mueller, M. S. Weiss, “*XDSAPP2.0*” *J. Appl. Crystallogr.* **2016**, *49*, 1085.

[47] W. Kabsch, “Integration, Scaling, Space-Group Assignment and Post-Refinement” *Acta Crystallogr. D Biol. Crystallogr.* **2010**, *66*, 133.

[48] D. Liebschner, P. V. Afonine, M. L. Baker, G. Bunkóczi, V. B. Chen, T. I. Croll, B. Hintze, L.-W. Hung, S. Jain, A. J. McCoy, N. W. Moriarty, R. D. Oeffner, B. K. Poon, M. G. Prisant, R. J. Read, J. S. Richardson, D. C. Richardson, M. D. Sammito, O. V. Sobolev, D. H. Stockwell, T. C. Terwilliger, A. G. Urzhumtsev, L. L. Videau, C. J. Williams, P. D. Adams, “Macromolecular Structure Determination Using X-Rays, Neutrons and Electrons: Recent Developments in Phenix” *Acta Crystallogr. Sect. Struct. Biol.* **2019**, *75*, 861.

[49] P. Emsley, B. Lohkamp, W. G. Scott, K. Cowtan, “Features and Development of Coot” *Acta Crystallogr. D Biol. Crystallogr.* **2010**, *66*, 486.

[50] G. N. Murshudov, A. A. Vagin, E. J. Dodson, “Refinement of Macromolecular Structures by the Maximum-Likelihood Method” *Acta Crystallogr. D Biol. Crystallogr.* **1997**, *53*, 240.

[51] E. F. Pettersen, T. D. Goddard, C. C. Huang, E. C. Meng, G. S. Couch, T. I. Croll, J. H. Morris, T. E. Ferrin, “UCSF ChimeraX : Structure Visualization for Researchers, Educators, and Developers” *Protein Sci.* **2021**, *30*, 70.

[52] J. D. Hunter, “Matplotlib: A 2D Graphics Environment” *Comput. Sci. Eng.* **2007**, *9*, 90.
